# Identification of the organic anion transporting polypeptides responsible for the hepatic uptake of the major metabolite of epyrifenacil, S‐3100‐CA, in mice

**DOI:** 10.1002/prp2.877

**Published:** 2021-10-07

**Authors:** Kengo Sakurai, Tomohiro Kuroda, Jun Abe, Hiroshi Toda, Sachiko Kitamoto

**Affiliations:** ^1^ Environmental Health Science Laboratory Sumitomo Chemical Co., Ltd. Osaka Japan

**Keywords:** active hepatic uptake, epyrifenacil, gender difference, herbicide, species difference, OATP

## Abstract

Epyrifenacil is a novel herbicide that acts as an inhibitor of protoporphyrinogen oxidase (PPO) and produces hepatotoxicity in rodents by inhibiting PPO. Our previous research revealed that the causal substance of hepatotoxicity is S‐3100‐CA, a major metabolite of epyrifenacil, and that human hepatocyte uptake of S‐3100‐CA was significantly lower than rodent one, suggesting less relevant to hepatotoxicity in humans. To clarify the species difference in the uptake of S‐3100‐CA, we focused on organic anion transporting polypeptides (OATPs) and carried out an uptake assay using human, rat, and mouse OATP hepatic isoforms‐expressing 293FT cells. As a result, all the examined OATPs were found to contribute to the S‐3100‐CA uptake, suggesting that the species difference was not due to the differences in selectivity toward OATP isoforms. When [^14^C]epyrifenacil was administered to mice, the liver concentration of S‐3100‐CA was higher in males than in females. Furthermore, when [^14^C]epyrifenacil was administered with OATP inhibitors, the liver/plasma ratio of S‐3100‐CA was significantly decreased by rifampicin, an Oatp1a1/Oatp1a4 inhibitor in mice, but not by digoxin, an Oatp1a4‐specific inhibitor. This result indicates that Oatp1a1, the predominant transporter in male mice, is the main contributor to the hepatic transport of S‐3100‐CA, and consequently to the gender difference. Moreover, we conclude that the species difference in the hepatic uptake of S‐3100‐CA observed in our previous research is not due to differences in the selectivity toward OATP isoforms but rather to the significantly higher expression of OATPs which mediate uptake of S‐3100‐CA in rodents than in humans.

## Graphical abstract

Epyrifenacilis a novel herbicide and its majormetabolite, S‐3100‐CA, was reported to cause hepatotoxicity in rodents via asignificant level of active uptake into hepatocytes. In this study, we have revealed that various OATPs mediate the uptake ofS‐3100‐CA by using OATP expressing cells. In addition, in vivo hepaticuptake of S‐3100‐CA was inhibited by rifampicin, an Oatp1a1/Oatp1a4 inhibitorin mice but not by digoxin, an Oatp1a4‐specific inhibitor, suggesting Oatp1a1 isthe predominant contributor of active uptake in mouse hepatocytes. Furthermore, from a considerationof the previous literatures, we knew that the expression levels of OATPs inlivers clearly mirror the gender and species difference in hepatic uptake ofS‐3100‐CA.

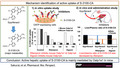

**Abbreviations**
AUC: area under the curve
DGX: digoxin
E_2_17βG: Estradiol 17β‐D‐glucuronide
E3S: Estrone 3‐sulfate
LSC: liquid scintillation counter
OATP: organic anion transporting polypeptide
PBS: phosphate‐buffered saline
PES: post‐extraction solid
PPIX: protoporphyrin IX
PPO: protoporphyrinogen oxidase
RIF: Rifampicin
RSV: rifamycin SV
SDS: sodium dodecyl sulfate
T1/2: half‐life
TLC: thin layer chromatography

## 1 INTRODUCTION

Epyrifenacil (ethyl[(3‐{2‐chloro‐4‐fluoro‐5‐[3‐methyl‐2,6‐dioxo‐4‐(trifluoromethyl)‐3,6‐dihydropyrimidin‐1(2*H*)‐yl]phenoxy}pyridin‐2‐yl)oxy]acetate, S‐3100) is a novel herbicide that acts as an inhibitor of protoporphyrinogen oxidase (PPO) in plants. PPO is involved in the synthesis of protoporphyrin IX (PPIX) which is essential for the biosynthesis of heme and chlorophyll in plants. Inhibition of PPO in plant cells causes accumulation of intermediate tetrapyrroles, including PPIX, which then leads to photoreactions that generate reactive oxygen and thereby to cell membrane destruction.^1^, ^2^ PPO is also present in mitochondria of mammals including rodents and humans, and catalyzes the production of heme which is necessary for the biosynthesis of red blood cells and various proteins such as metabolic enzymes. Thus, it is essential for vital activity in all mammals. It is reported that PPO inhibitors can cause toxicity, especially in PPO‐generating organs such as the liver, and occasionally induce hepatocellular tumors as a consequence of hepatotoxicity.^3^, ^4^, ^5^ In the case of epyrifenacil, a 90‐day subchronic toxicity study showed that epyrifenacil produced hepatocyte injury in mice and rats but not in dogs.^6^ Since there was a significant species difference, further investigation of hepatotoxicity was considered necessary for the safety assessment of epyrifenacil's hepatotoxicity in humans.

Previously, we conducted several in vitro mechanistic assays and in vivo metabolism experiments for the purpose of evaluating the toxicity of epyrifenacil in humans and elucidating species differences in sensitivity and exposure. In the in vitro metabolism study, the ester bond of epyrifenacil was rapidly hydrolyzed to form a carboxylic acid metabolite named S‐3100‐CA in mouse, rat, and human liver microsomes.^6^ In addition, the in vivo metabolism study also confirmed that S‐3100‐CA was the most abundant metabolite in both the liver and plasma of rats, whereas epyrifenacil was not detected in these samples (Sakurai et al., unpublished). In the PPO inhibition study, S‐3100‐CA showed comparable inhibitory activity to that of the parent epyrifenacil.^6^ From these results, it was proposed that the hepatotoxic metabolite of epyrifenacil was S‐3100‐CA. For human safety assessment, the PPO inhibition assay of the human mitochondrial fraction revealed that the inhibitory activity of S‐3100‐CA against mitochondrial PPO was 10 times higher in mice than in humans. In addition, the amount of S‐3100‐CA taken up by mouse hepatocytes was 6–13 times higher than that taken up by human hepatocytes. From these results, it was suggested that the hepatotoxicity potential of epyrifenacil is much lower in humans than in rodents.

We focused on one of these two key factors affecting species difference in hepatotoxicity: the mechanism of S‐3100‐CA accumulation in the liver of rodents. We speculated that transporters should be involved in active hepatic uptake because (1) the major metabolite S‐3100‐CA likely exists as a carboxylic anion in vivo and (2) the gender difference in hepatic uptake of S‐3100‐CA was observed in rodents, i.e., the amount of uptake was higher in males than in females. Furthermore, (3) the gender difference in urinary excretion of S‐3100‐CA was also observed in the rat metabolism study. All the above observations are well in line with the metabolic fate of some known substrates of organic anion transporting polypeptides (OATPs).^7^, ^8^, ^9^ Therefore, we focused on OATPs that exist in both liver and kidney and have expression levels that differ between male and female rodents.

OATPs are reported to transport anionic compounds into tissues, impacting hepatic pharmacokinetics.^10^, ^11^, ^12^ About 14% of transporters in mouse liver are OATPs, and Oatp1a1, Oatp1a4, Oatp1b2, and Oatp2b1 are the major isoforms.^13^ Notably, there is a gender difference in the expression levels of some OATPs in mice, Oatp1a1 and Oatp1a4 are highly expressed in males and females, respectively.^14^ OATPs are also major transporters in human liver.^15^, ^16^ OATP1B1, OATP1B2, and OATP2B1 are the major human OATP isoforms. Although the involvement of OATPs in the hepatic uptake of S‐3100‐CA was suggested from the previous studies, it was still unknown which of the transporter isoform(s) are the main contributors to S‐3100‐CA transport. Furthermore, it is unclear whether the species difference in the hepatic uptake of S‐3100‐CA observed in previous hepatocyte assays can also be explained by the involvement of OATPs in this process.

In this report, we investigate the hepatic uptake of S‐3100‐CA by OATPs to elucidate the mechanism of hepatic uptake in rodents and to explain the species difference in hepatic uptake between rodents and humans. To identify transporter isoforms, each OATP seen in rodent and human livers was transiently transfected into 293FT cells, and the uptake of S‐3100‐CA by these transporters was measured. Moreover, to confirm the impact of transporters in mouse pharmacokinetics, we performed an in vivo study of radiolabeled epyrifenacil co‐administered with OATP inhibitors.

## 2 MATERIALS AND METHODS

### 2.1 Chemicals

**FIGURE 1:**
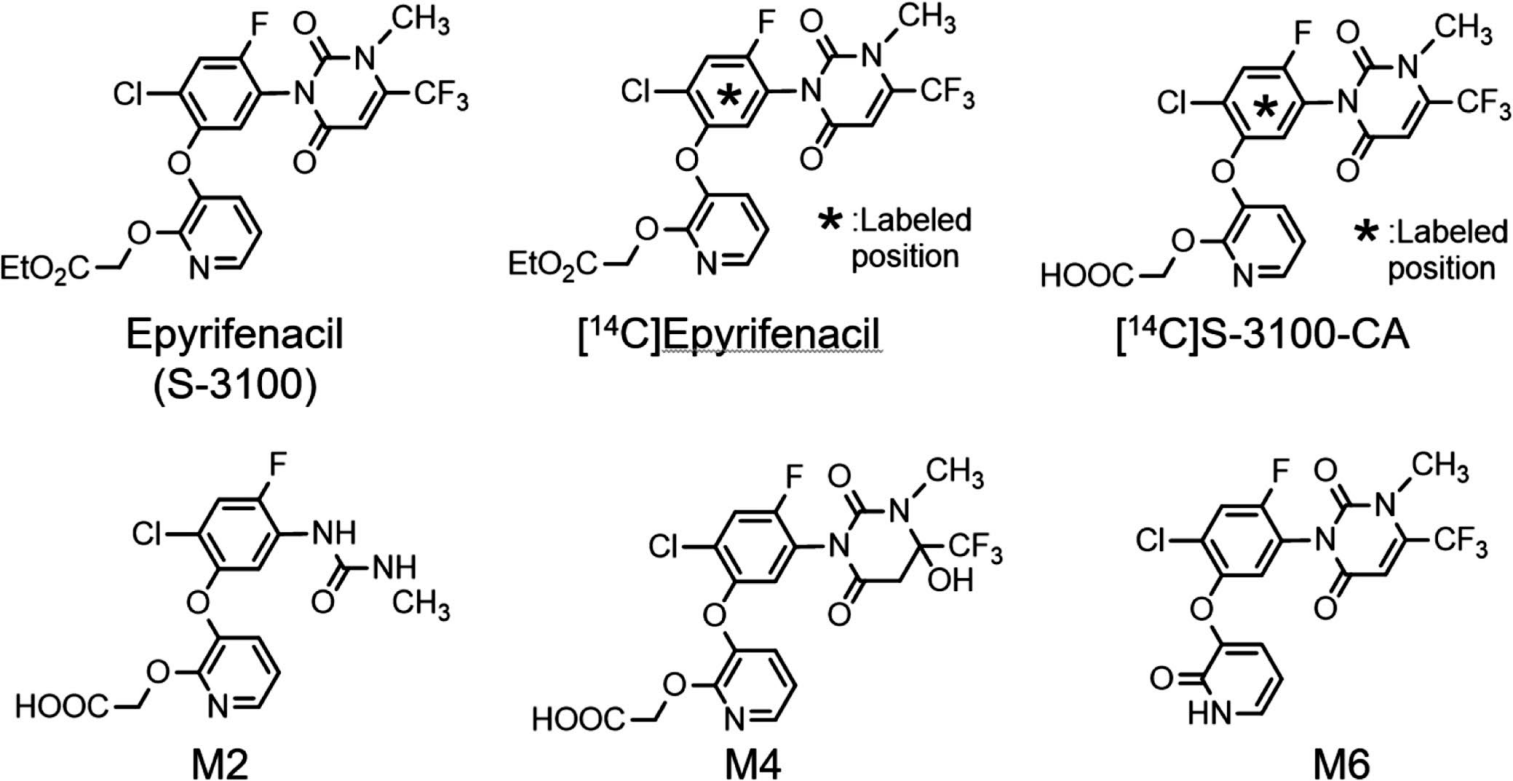
Chemical structures of radiolabeled, non‐radiolabeled epyrifenacil, and metabolites of epyrifenacil

Epyrifenacil (S‐3100), phenyl‐^14^C‐labeled epyrifenacil ([^14^C]epyrifenacil), and phenyl‐^14^C‐labeled S‐3100‐CA ([^14^C]S‐3100‐CA) were synthesized in our laboratory. The chemical purity of epyrifenacil was 99.8%. The radiochemical purities of [^14^C]epyrifenacil and [^14^C]S‐3100‐CA were 99.5% and 97.8%, respectively, with the specific activity of each compound being 4.26 GBq/mmol. Authentic metabolite standards of S‐3100‐CA (M1), M2, M4, and M6 were also synthesized in our laboratory. The structures of these compounds are shown in Figure 1. [estradiol‐6,7‐^3^H(N)]Estradiol 17β‐D‐glucuronide ([^3^H]E_2_17βG) and [6, 7‐^3^H(N)]estrone 3‐sulfate ([^3^H]E3S) were purchased from PerkinElmer, Inc. (Waltham, MA, USA). The specific activity of [^3^H]E_2_17βG and [^3^H]E3S was 1713 and 1820 GBq/mmol, respectively. Rifampicin (RIF) was purchased from FUJIFILM Wako Pure Chemical Co., rifamycin SV (RSV) was purchased from Cayman Chemical Company, and digoxin (DGX) was purchased from Tokyo Chemical Industry Co., Ltd. Methylcellulose #400 was purchased from Nacalai Tesque, Inc. Other chemicals were reagent grade.

### 2.2 Measurement of radioactivity

Radioactivity was measured by a liquid scintillation counter (LSC, Tri‐Carb^®^ 3110TR, PerkinElmer, Inc.). The radioactivity in liquid samples (i.e., cell lysate, tissues and plasma extracts) was counted directly after mixing the samples with 10 ml Emulsifier Scintillator Plus™ (PerkinElmer, Inc.) in low potassium glass vials. Blood, plasma, and tissue samples were solubilized with 3 M potassium hydroxide solution (approximately 1 ml/vial) in a glass vial, then bleached with 30% hydrogen peroxide solution, and finally mixed with 10 ml of Hionic‐Fluor™ (PerkinElmer, Inc.) for LSC analysis.

### 2.3 Chromatographic procedures

Precoated silica gel 60 F254 chromatoplate (20 × 20 cm, 0.25‐ or 0.5‐mm layer thickness, Merck KGaA) was used for two‐dimensional thin layer chromatography (2D‐TLC) analysis. The solvent systems were ethyl formate/formic acid (95/5, v/v) for the first dimension, and ethyl acetate/acetic acid (95/5, v/v) for the second dimension. The radioactivity on the TLC plates was detected by autoradiography using an imaging plate processed with a fluoro‐image analyzer (FLA‐9500, GE Healthcare). The identity of the metabolites was confirmed by co‐chromatography in comparison with authentic metabolite standards.

### 2.4 Cell Culture and transfection of OATPs

293FT cells were purchased from Thermo Fisher Scientific, Inc.. The cells were grown in Dulbecco's modified Eagle medium (supplemented with 4.5 g/L glucose and 2 mM L‐glutamine) at 37°C, 5% CO_2_, and 95% humidity. The cells were passaged 3 times per week by harvesting cells using trypsin and plating at a density of 1.0 × 10^4^ cells/cm^2^ in a 10 cm cell culture plate for maintenance culture. The 293FT cells were detached using trypsin and re‐seeded at a density of 2.5 × 10^4^ cells/cm^2^ in BioCoat™ Collagen I 6‐well plates (Corning Inc.) for transfection and then treated with 1.2 μg of plasmid vector and 3.2 μl of Lipofectamine™ 2000 Transfection Reagent (Thermo Fisher Scientific, Inc.) according to the manufacturer's instructions. After a 3‐h treatment, the transfection medium was replaced with a fresh cell culture medium and incubated at 37°C with 95% humidity and 5% CO_2_ until ready for the assay.

### 2.5 Uptake assay for radiolabeled substrates via OATPs

Transport experiments were conducted approximately 24 h post‐transfection. The experiments were initiated by aspirating culture medium and addition of pre‐warmed radiolabeled substrate solution (final 1.0% of acetonitrile or 0.9% of ethanol in phosphate‐buffered saline (PBS[−])) to each well. After a 10‐min incubation, cells were washed twice with 1 ml of ice‐cold PBS(−) and lysed with 700 μl of 1.0 M NaOH for 3 h or more at room temperature. The lysate was transferred to a 1.5‐ml tube, and neutralized with 350 μl of 2.0 M HCl and 350 μl of 2.0% sodium dodecyl sulfate (SDS) solution. From the lysate, 1.0 ml was transferred to a vial containing 10 ml of Emulsifier Scintillator Plus to measure the radioactivity in the LSC. The protein concentration of the cell lysate was also measured using a Pierce™ BCA Protein Assay Kit (Thermo Fisher Scientific, Inc.), which was used to normalize, according to the cell number, the radioactivity in the cell lysate extract. The protein assay was performed in duplicate. Each sample type was assayed in triplicate wells, and each run was performed twice. When the difference in mean uptake between OATP expressing cells and mock cells was more than 2 times, the OATP was judged to be involved in transportation of the substrate. The uptake assay was repeated with OATP inhibitors, RIF, RSV, and DGX. The method was generally similar to the uptake assay described above, except that the substrate solution contained an inhibitor (i.e., at final concentration of 1, 10, and 100 μM of RIF or RSV, or 10 and 100 μM of DGX, with final concentration of dimethyl sulfoxide, 1.0%). Each sample type was assayed in triplicate wells. The inhibition assay was performed only once because the fold‐increase of the uptake by the expression of OATPs was comparable among the runs, and thus, it was concluded that the expression of OATPs is stable.

### 2.6 Treatment of animals

Crl:CD1(ICR) mice at the age of 7 weeks were purchased from Charles River Laboratories Japan, Inc., Hino Breeding Center. The mice at the age of 8 or 9 weeks were dosed after completing a 7‐day quarantine period and an additional 0‐ to 7‐day acclimatization period. All animal experiments were conducted in accordance with the Guidance for the Care and Use of Laboratory Animals in our laboratory, which corresponds to the Guidelines for Proper Conduct of Animal Experiments (Science Council of Japan). Animals were maintained under constant environmental conditions: room temperature, 21−25°C; relative humidity, 55% ±15%; ventilation, 10 air exchanges per hour; and artificial lighting, from 8:00 am to 8:00 pm. The animals were provided with pelleted diet and water ad libitum. All tested animals showed normal weight gain and no abnormal clinical symptoms during the 7–14 days of quarantine and acclimatization, and their body weights were 31.95−40.20 g for males and 22.39−30.43 g for females when dosed. All animals were euthanized by bleeding from the abdominal artery under anesthesia.

### 2.7 Preparation of dosing solutions

[^14^C]Epyrifenacil was isotopically diluted with the unlabeled test compound to adjust the specific activity to 5.00 MBq/mg. After evaporation of the solvent under nitrogen gas flow, labeled material was suspended in 0.5% (w/v) methylcellulose solution at a concentration of 1 mg/5 ml by grinding in a mortar. The suspension was stored frozen until the start of dosing. The radiochemical purity of the dosing suspension was analyzed by radio‐HPLC and was confirmed to be >99%. For an inhibitory study of the in vivo experiment, dosing solutions of inhibitors (RIF or DGX) were prepared. RIF was weighed and suspended in 0.5% (w/v) methylcellulose solution at concentration of 40 mg/ml by grinding in a mortar, and DGX was dissolved in physiological saline at a concentration of 40 mg/ml. These dosing suspensions were stored in a refrigerator until dosing commenced.

### 2.8 Blood ^14^C‐concentrations study

Two mice per sex at 8 weeks old were given a single oral dose of [^14^C]epyrifenacil at 1 mg/kg. Approximately 0.06–0.08 g of blood sample was periodically taken from the saphenous vein at 1, 2, 4, and 8 h after administration, and transferred to vials to measure the radioactivity in whole blood. The half‐life (*T*_1/2_) of radioactivity in blood was calculated from the blood concentrations at 4–8 h in male mice and 1–8 h in female mice, by linear regression analysis with a logarithmic transformation of each value. AUC_0‐∞_ was calculated as the sum of AUC_0‐8 h_ and AUC_8 h‐∞_. AUC_0‐8 h_ was obtained using a trapezoidal rule, and AUC_8 h‐∞_ was calculated with a mono‐exponential equation describing the ^14^C‐disappearance phase.

### 2.9 ^14^C‐plasma, liver, and kidney distribution study

Three mice per sex at 8 weeks old were given a single oral dose of [^14^C]epyrifenacil at 1 mg/kg with or without co‐administration of OATP inhibitors. Approximately 5 ml per kg body weight of a dosing suspension of RIF or DGX was orally administered 1 h before the administration of [^14^C]epyrifenacil. A dosing suspension of 2.5 ml DGX per kg body weight was also intraperitoneally administered immediately before administration of [^14^C]epyrifenacil. At 2 h after administration of [^14^C]epyifenacil, the animals were euthanized by whole blood collection from the abdominal aorta under anesthesia with isoflurane. The sampling time point was selected as the time around *T*_max_ based on the result of the blood ^14^C‐concentration study. Then, the liver and kidney were dissected out, and the amount of ^14^C was measured. Aliquots of heparinized blood samples were separated into blood cells and plasma by centrifugation at 2000*g* for 10 min at 4°C. The total weight of the liver and kidney was determined for each animal. Two aliquots of plasma and tissue samples (approximately 0.05–0.1 g) were weighed in a glass vial, and the radioactivity was measured.

### 2.10 Metabolite analysis

Approximately 0.3 g of liver, kidney, and plasma samples were pooled, respectively, for each dose and gender to obtain samples for analysis. Pooled liver, kidney, and plasma (ca. 1 g each) samples were homogenized with an approximately 3‐fold volume (v/w) of methanol using the mixer mill MM400 (5 min, 25 cycles/second, Retsch GmbH) and centrifuged (10 000*g*, 5 min, 4°C) to separate the supernatants from the residues. The extraction was repeated one additional time. The supernatants obtained were pooled and labeled liver, kidney, and plasma extracts. These samples were concentrated and then subjected to TLC analysis. For calculation of extraction efficacy, the post‐extraction solids (PESs) from the tissues and plasma extracts were sampled in low potassium glass vials. PESs were solubilized with 3 M potassium hydroxide solution (approximately 1 ml), then bleached with 30% hydrogen peroxide solution, and finally mixed with 10 ml of Hionic‐Fluor to measure the radioactivity.

### 2.11 Data analysis

Average, standard deviation, standard error, and other statistical metrics were calculated using Microsoft Excel 2016.

## 3 RESULTS

### 3.1 Establishment of OATP expressing cells

We established OATP expressing cells using the method above and confirmed their expression of OATPs and the functionality of each OATP by measuring the uptake of two well‐known OATP substrates. OATP expressing cells were assayed with E_2_17βG and E3S and the result was used to calculate the uptake ratios which represent the fold increase in uptake relative to uptake by cells transfected with the control plasmid (mock cells) (Table 1). All the uptakes of E_2_17βG and E3S by OATP expressing cells were more than twice as large as those by mock cells except for the uptake of E3S via mouse/rat Oatp1a4 (1.1–1.8 fold) and the uptake of E_2_17βG via human OATP2B1 (1.3 fold) when considered with the mean of 2 runs. Those OATPs with increased uptakes was confirmed to be involved in transportation of each substrate. Especially, the uptake of E_2_17βG by human OATP1B1, mouse Oatp1a1, and rat Oatp1a1, and uptake of E3S by OATP1B1 were remarkably high (>50 times higher than in mock cells).

**TABLE 1:** Ratio of uptake amount of S‐3100‐CA into various OATP expressing cells relative to the uptake into mock cells after a 10‐min incubation

Mock cell	E_2_17βG		E3S		S‐3100‐CA	
	Run_1	Run_2	Run_1	Run_2	Run_1	Run_2
	1.0	1.0	1.0	1.0	1.0	1.0
Human						
OATP1B1	48.2	55.6	69.9	81.9	5.7	6.2
OATP1B3	19.3	12.9	2.7	3.3	3.1	5.1
OATP2B1	1.9	0.6	16.2	1.9	2.6	1.9
Mouse						
Oatp1a1	84.9	62.2	23.4	34.4	3.3	4.3
Oatp1a4	3.7	3.3	1.1	1.1	8.1	4.2
Oatp1b2	8.3	14.7	23.8	19.7	11.7	6.2
Oatp2b1	4.1	2.0	2.9	3.9	2.4	4.0
Rat						
Oatp1a1	59.1	65.4	28.6	13.6	11.7	8.7
Oatp1a4	6.1	10.4	2.2	1.4	5.0	4.1
Oatp1b2	27.4	43.2	10.0	4.7	6.6	8.4

Note

Values are presented as individual values of the data from two runs. Each value in a single run represents mean of 3 replicates.

### 3.2 S‐3100‐CA uptake into OATP expressing cells

Using the constructed OATP expressing cells, we evaluated S‐3100‐CA uptake. The uptake ratios with all OATP expressing cells were at least twice that with mock cells (Table 1). These OATPs were judged to be involved in transportation of S‐3100‐CA. The mean uptake ratios with OATP expressing cells varied among the isoforms but all above 2.0, suggesting that S‐3100‐CA is transported via various OATP isoforms without typical selectivity toward isoforms.

### 3.3 Inhibition of S‐3100‐CA uptake by OATP inhibitors

**FIGURE 2:**
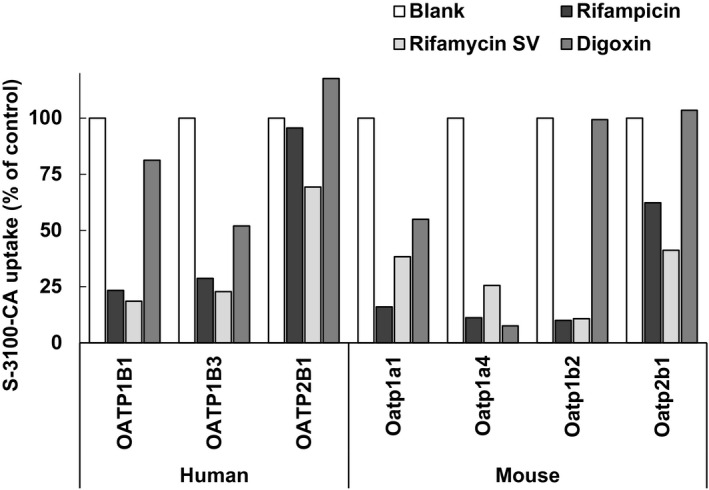
Inhibitory effect of rifampicin, rifamycin SV, and digoxin at 100 μM for various OATPs that mediate the uptake of S‐3100‐CA. All data are indicated as the mean value of 3 replicates in a single run

**FIGURE 3:**
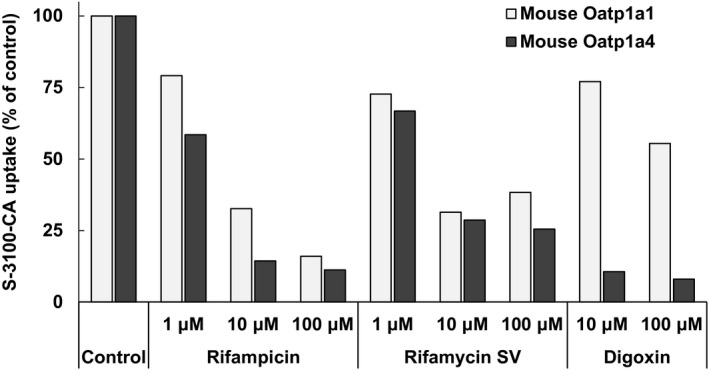
Concentration‐dependent inhibition of the main hepatic OATPs that mediate the uptake of S‐3100‐CA in mice. All data are indicated as the mean value of 3 replicates in a single run

Inhibition of uptake of S‐3100‐CA via OATPs was also evaluated using OATP inhibitors (Figure 2). The addition of RIF eliminated the uptake to less than 40% of that by vehicle‐treated cells via various OATP, i.e., mouse Oatp1a1, Oatp1a4, and Oatp1b2, and human OATP1B1 and OATP1B3. Similarly, RSV inhibited uptake via all OATPs other than human OATP2B1. On the other hand, DGX inhibited uptake via only mouse Oatp1a4 at a high rate. DGX also inhibited uptake via mouse Oatp1a1 and human OATP1B3, but it was less potent (>50% vs. control). For mouse Oatp1a1 and Oatp1a4, inhibitory effect of these inhibitors was further examined at various concentrations (Figure 3). All three chemicals inhibited the uptake of S‐3100‐CA via mouse Oatp1a1 and Oatp1a4 in a concentration‐dependent manner; however, the inhibition of uptake via Oatp1a1 by DGX was weak.

### 3.4 Kinetic parameters of uptake in mice dosed with [^14^C]epyrifenacil

^14^C‐Concentrations in blood within 8 h after a single oral administration of [^4^C]epyrifenacil to male and female mice at doses of 1 mg/kg were determined (Table 2). ^14^C‐Concentrations in blood rapidly increased in both males and females to reach maximum blood concentrations (*C*_max_) of 0.040 and 0.100 µg eq. of epyrifenacil/g (hereinafter referred to as ppm) at 4 and 1 h after administration, respectively. After the maximum concentration time (*T*_max_), concentrations of radioactivity decreased in blood rapidly. The mean terminal elimination half‐lives (T_1/2_) were determined to be 8 h in both male and female mice. The area under the curve (AUC_0‐∞_) in blood was 0.560 and 1.116 µg eq. of epyrifenacil·h/g in male and female mice, respectively. Blood ^14^C‐concentration was relatively high in females at all sampling time points. AUC_0‐∞_ was also higher in females despite the similar *T*_1/2_ between males and females. Accordingly, we set the time point of dissection at 2 h after administration, which accounts for the high ^14^C exposure in both males and females.

**TABLE 2:** ^14^C‐Concentrations and pharmacokinetic parameters of ^14^C in blood after a single oral administration of [^14^C]epyrifenacil at 1 mg/kg to male and female mice

Time after administration (h)		^14^C‐Concentration (µg eq. of epyrifenacil/g)			
		Male		Female	
		Animal_1	Animal_2	Animal_1	Animal_2
1		0.030	0.030	0.127	0.073
2		0.031	0.034	0.111	0.064
4		0.028	0.051	0.076	0.060
8		0.024	0.033	0.045	0.061
*T*_max_ (h)		4		1	
*C*_max_ (μg eq. of epyrifenacil/g)		0.040		0.100	
*T*_1/2_ (h)		8		8	
AUC (µg eq. of epyrifenacil·h/g)	(0–8 h)	0.246		0.542	
	(0–∞)	0.560		1.116	

Note

^14^C‐Concentration data are presented as the individual values of two mice. Pharmacokinetic parameters (*T*_max_, *C*_max_, and AUC) were calculated from the mean values of ^14^C‐concentration in blood.

### 3.5 ^14^C‐Distribution in plasma, liver, and kidney

^14^C‐Concentrations were determined in plasma, liver, and kidney at 2 h after single oral administration of [^14^C]epyrifenacil to male and female mice (Table 3). In mouse plasma, liver, and kidney, no remarkable gender‐related differences in ^14^C‐concentration were observed. Notably, the concentration in liver was 6.763 ± 0.1014 and 5.136 ± 1.1225 ppm for males and females, respectively, which was somewhat higher than concentrations in plasma and kidney. The liver‐to‐plasma concentration ratios were ca. 73 and 35 in males and females, respectively.

**TABLE 3:** ^14^C‐Concentrations in plasma, liver, and kidney at 2 h after a single oral administration of [^14^C]epyrifenacil at 1 mg/kg to male and female mice

	^14^C‐Concentration (μg eq. of epyrifenacil/g tissue)^a^					
	Plasma		Liver		Kidney	
	Male	Female	Male	Female	Male	Female
Epyrifenacil (1 mg/kg p.o.)	0.092 ± 0.0066	0.147 ± 0.0642	6.763 ± 0.1014	5.136 ± 1.1225	0.124 ± 0.0180	0.190 ± 0.0511
	(NA^b^)	(NA)	(73)	(35)	(1.3)	(1.3)
Epyrifenacil (1 mg/kg p.o.) + Rifampicin (200 mg/kg p.o.)	0.151 ± 0.0791	0.217 ± 0.1551	1.671 ± 1.2478	0.880 ± 0.4160	0.144 ± 0.0794	0.130 ± 0.0548
	(NA)	(NA)	(11)	(4.0)	(0.96)	(0.60)
Epyrifenacil (1 mg/kg p.o.) + Digoxin (200 mg/kg p.o.)	0.017 ± 0.0064	0.054 ± 0.0118	1.647 ± 0.7217	2.344 ± 0.4524	0.044 ± 0.0051	0.086 ± 0.0096
	(NA)	(NA)	(97)	(43)	(2.6)	(1.6)
Epyrifenacil (1 mg/kg p.o.) + Digoxin (100 mg/kg i.p.)	0.083 ± 0.0027	0.082 ± 0.0574	5.527 ± 1.3453	3.390 ± 1.9870	0.140 ± 0.0527	0.184 ± 0.0360
	(NA)	(NA)	(67)	(41)	(1.7)	(2.2)

Note

Values are presented as the mean ± standard deviation of the data from three mice.

^a^ Values in parenthesis are the tissue/plasma ratio of ^14^C.

^b^ Not applicable.

### 3.6 ^14^C‐Distribution in plasma and tissues with co‐administration of OATP inhibitors

^14^C‐Concentrations in plasma, liver, and kidney at 2 h after a single oral administration of [^14^C]epyrifenacil with co‐administration of OATP inhibitors are presented in Table 3. After a single oral co‐administration of RIF at dose of 200 mg/kg, ^14^C‐concentration in liver was found to be 1.671 and 0.880 ppm for males and females, respectively, which was lower than that of mice treated with [^14^C]epyrifenacil alone, and the ^14^C‐concentration of plasma and kidney of mice co‐administrated with RIF was not significantly different with that of mice treated with [^14^C]epyrifenacil alone. As a result, the ^14^C‐concentration in liver was ca. 11 and 4 times higher than in plasma in males and females, respectively.

On the other hand, after a single oral co‐administration of DGX at dose of 200 mg/kg, ^14^C‐concentration in plasma, liver, and kidney was lower than that of mice treated with [^14^C]epyrifenacil alone. Compared to the ^14^C‐concentration in plasma, the ^14^C‐concentration in liver was ca. 97 times higher in males and ca. 43 times higher in females. It was confirmed that the liver‐to‐plasma concentration ratios were not significantly changed by oral administration of DGX. Intraperitoneal co‐administration of DGX at a dose of 100 mg/kg did not notably change both ^14^C‐concentrations and liver/plasma concentration ratios.

### 3.7 Quantification of metabolites of in plasma, liver, and kidney

**FIGURE 4:**
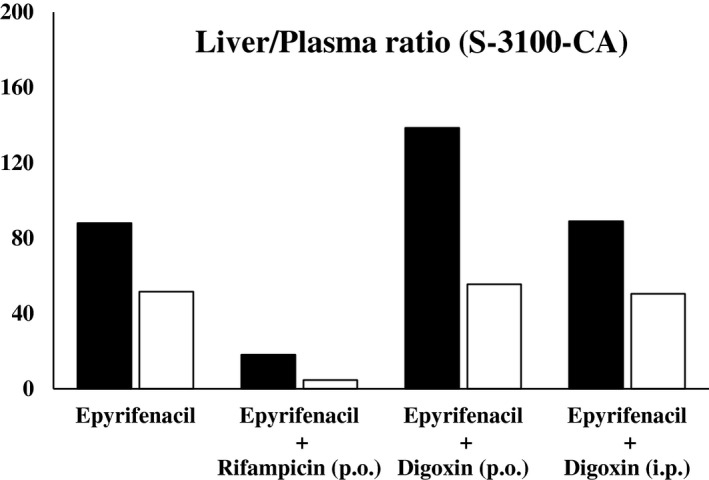
Liver/plasma ratio of S‐3100‐CA concentrations at 2 h after a single oral administration of [phenyl‐^14^C]epyrifenacil at 1 mg/kg to male (filled bar) and female (open bar) mice with or without co‐administration of OATP inhibitors. Values were obtained by the analysis of pooled samples from three animals

Concentrations (μg eq. of epyrifenacil/g or ppm) of metabolites in plasma, liver, and kidney were determined by TLC (Table 4), and the tissue/plasma ratios of S‐3100‐CA were calculated (Figure 4). In the plasma, liver, and kidney extracts, the metabolic profiles were almost the same, and 4 metabolites which identified in the rat metabolism study, S‐3100‐CA (M1), M2, M4, and M6 were also detected (Sakurai et al., unpublished). The major metabolite in liver was S‐3100‐CA, accounted for over 70% of ^14^C in all dose groups. In line with the higher total ^14^C in liver, S‐3100‐CA concentration was also high in liver compared to plasma and kidney, and the liver/plasma ratios of S‐3100‐CA were 88 in males and 52 in females dosed epyrifenacil without inhibitors. When RIF was co‐administrated with [^14^C]epyrifenacil, S‐3100‐CA was still higher in liver (1.371 and 0.660 ppm for males and females, respectively), and consequently, liver/plasma ratio was lowered to 18 and 5 for males and females, respectively. In mice with oral co‐administration of DGX, concentrations of S‐3100‐CA in liver, kidney and plasma were lower than that of mice without inhibitor. Although, when DGX was intraperitoneally co‐administrated with [^14^C]epyrifenacil, concentrations of S‐3100‐CA in liver, kidney and plasma were not significantly different from that in mice which was administrated with [^14^C]epyrifenacil alone. The liver/plasma ratio of DGX co‐administrated mice was not decreased, which accounted the ratios of 89–139 and 50–56 in males and females, respectively.

**TABLE 4:** Concentrations of metabolites in plasma, liver, and kidney at 2 h after single oral administration of [^14^C]epyrifenacil at 1 mg/kg to male and female mice

^14^C‐Concentration (μg eq. of epyrifenacil/g plasma or tissue)						
Metabolite	Plasma		Liver		Kidney	
	Male	Female	Male	Female	Male	Female
[^14^C]Epyrifenacil (1 mg/kg p.o.)						
S‐3100‐CA (M1)	0.054	0.068	4.740	3.525	0.055	0.096
M2	0.020	0.055	0.024	0.011	0.004	0.002
M4	0.003	N.D.	1.033	0.858	0.009	0.023
M6	0.003	0.015	0.012	0.006	0.008	0.004
Others	0.011	0.006	0.222	0.071	0.035	0.050
Subtotal	0.090	0.145	6.030	4.471	0.111	0.175
Unextractable	0.002	0.003	0.732	0.665	0.013	0.015
Total	0.092	0.147	6.763	5.136	0.124	0.190
[^14^C]Epyrifenacil (1 mg/kg, p.o.) + Rifampicin (200 mg/kg, p.o.)						
S‐3100‐CA (M1)	0.076	0.143	1.371	0.660	0.073	0.050
M2	0.035	0.016	N.D.	N.D.	0.001	N.D.
M4	0.004	0.022	0.172	0.129	0.022	0.015
M6	N.D.	N.D.	N.D.	N.D.	0.001	N.D.
Others	0.035	0.033	0.034	0.037	0.025	0.044
Subtotal	0.149	0.215	1.576	0.827	0.123	0.109
Unextractable	0.002	0.003	0.095	0.054	0.022	0.022
Total	0.151	0.217	1.671	0.880	0.144	0.130
[^14^C]Epyrifenacil (1 mg/kg, p.o.) + Digoxin (200 mg/kg, p.o.)						
S‐3100‐CA (M1)	0.009	0.029	1.187	1.587	0.024	0.035
M2	0.006	0.012	N.D.	0.004	N.D.	N.D.
M4	N.D.	N.D.	0.157	0.291	0.005	0.007
M6	N.D.	N.D.	0.005	0.004	N.D.	N.D.
Others	0.002	0.013	0.191	0.315	0.009	0.020
Subtotal	0.016	0.053	1.539	2.200	0.038	0.061
Unextractable	0.000	0.001	0.108	0.143	0.006	0.025
Total	0.017	0.054	1.647	2.344	0.044	0.086
[^14^C]Epyrifenacil (1 mg/kg, p.o.) + Digoxin (100 mg/kg, i.p.)						
S‐3100‐CA (M1)	0.043	0.045	3.830	2.260	0.058	0.091
M2	0.013	0.009	N.D.	0.002	0.002	0.001
M4	N.D.	N.D.	0.651	0.553	0.014	0.030
M6	0.004	0.003	0.013	0.005	0.001	0.002
Others	0.021	0.025	0.550	0.103	0.043	0.023
Subtotal	0.081	0.081	5.044	2.924	0.118	0.147
Unextractable	0.002	0.001	0.482	0.467	0.022	0.037
Total	0.083	0.082	5.527	3.390	0.140	0.184

## 4 DISCUSSION

In this study, we investigated the involvement of OATPs in the hepatic uptake of S‐3100‐CA to elucidate the mechanism of hepatic uptake in rodents and to explain the species difference between rodents and humans. It is commonly recognized that various OATPs are expressed in the livers of mammals, including humans, rats, and mice.^13^, ^16^ In this study, we used E_2_17βG and E3S as the positive control substances for the OATP expressing cells established, and confirmed their uptake by almost all OATP expressing cells. These observations about OATP‐mediated uptake of E_2_17βG and E3S by human and rodent OATP isoforms agreed with those in previous articles.^17^, ^18^, ^19^, ^20^, ^21^, ^22^, ^23^, ^24^, ^25^ As exceptions, uptake of E3S by mouse/rat Oatp1a4 and that of E_2_17βG by human OATP2B1 were not large (not more than 2‐fold) compared to the uptake of these substrates by mock cells. Among them, it has been reported that E3S was not transported via mouse Oatp1a4 and that E_2_17βG was not transported via human OATP2B1.^26^, ^27^, ^28^ Regarding the uptake of E3S by rat Oatp1a4, no previous report is available; however, another substrate E_2_17βG was transported significantly via rat Oatp1a4, suggesting that these exceptions of less uptake were due to the difference of substrate specificity of these OATP isoforms. From these results, we concluded that all OATPs were expressed in transfected cells as expected, and worked to transport the substrates appropriately.

S‐3100‐CA was also taken up by all OATPs examined in this study. The uptake ratios versus mock cells were at least 2.2, thus it was suggested that S‐3100‐CA is transported via various OATPs without typical selectivity toward the isoforms. Although the uptake ratios varied among the OATP isoforms, the differences in the kinetics of S‐3100‐CA uptake via OATP isoforms in hepatocytes or intact liver was not directly indicated, because these results were not corrected by expressed protein levels. Moreover, the results showed no obvious differences in the uptake potential between predominant hepatic OATP isoforms (i.e., Oatp1a1, Oatp1a4, Oatp1b2, Oatp2b1 in mice and OATP1B1, OATP1B3, OATP2B1 in humans). As mentioned above, significant species differences were observed in the hepatic uptake of S‐3100‐CA.^6^ With the currently‐available data, the reason of the species differences was considered not due to the selectivity of S‐3100‐CA toward OATP isoforms.

Prior to the OATP inhibition experiment in vivo, the selectivity of the known OATP inhibitors (RIF, RSV, and DGX) in rats and humans was determined for mouse isoforms because such information has not been well reported. As a result, DGX was revealed to be a selective inhibitor of Oatp1a4 in mice. On the other hand, RIF and RSV inhibited mouse Oatp1a1, mouse Oatp1a4, human OATP1B1, and human OATP1B3. These inhibitory effects on mouse OATPs increased in a dose‐dependent manner and reached more than 50% at 10 μM. The selectivity of the inhibitory effects of these inhibitors was similar to that for the rat OATPs reported previously.^29^ With these results, we decided to use RIF and DGX in the in vivo study as mouse OATP inhibitors.

After administering [^14^C]epyrifenacil to mice, we measured ^14^C‐ and metabolite concentrations to confirm that the significant hepatic uptake and its gender differences observed in in vitro hepatocyte assay are reproduced in vivo. ^14^C‐Concentrations in plasma and kidney of [^14^C]epyrifenacil dosed mice were slightly higher in females than in males. On the other hand, the concentration in liver was higher in males. As a result, the ratio of liver to plasma ^14^C‐concentration was ca. 73 and 35 for males and females, respectively. There was a gender‐related difference in liver/plasma ratio. A metabolite analysis of these fractions identified S‐3100‐CA as the predominant metabolite with high and concomitant gender difference in liver/plasma ratio; the liver/plasma ratio of S‐3100‐CA was 88 and 52 for males and females, respectively. This result was consistent with that of the hepatocyte uptake assay,^6^ S‐3100‐CA uptake was high and associated with a gender difference. Some studies revealed a gender difference in the expression level of Oatp1a1 and Oatp1a4 in mouse liver, with Oatp1a1 expression approximately doubled in male mouse liver and Oatp1a4 expression doubled in female mouse liver.^14^ These gender differences in the OATP expression were reported to cause the species differences in the hepatic uptake for some OATP substrates,^7^, ^9^ thus it was suggested that the higher hepatic uptake of S‐3100‐CA in male mice was also due to the gender difference in Oatp1a1 expression levels.

In order to investigate the contribution of Oatp1a1 and Oatp1a4 to species differences in the kinetics in vivo, OATP inhibitors were co‐administered with [^14^C]epyrifenacil. Our in vitro experiment revealed that RIF inhibited mouse Oatp1a1 and Oatp1a4, and that DGX inhibited Oatp1a4 specifically. When [^14^C]epyrifenacil and RIF were co‐administered, ^14^C‐concentration in liver became significantly lower than in the livers of control mice without RIF, whereas ^14^C‐concentration in plasma was slightly increased, resulting in lower liver/plasma ratio of 11 and 4 for males and females, respectively. Co‐administration with RIF also decreased S‐3100‐CA concentration in liver; when compared to the mice without inhibitors, mice with inhibitors had a significantly lower concentration in liver and relatively higher concentration in plasma, and consequently, the liver/plasma ratio was lowered to 18 and 5 for males and females, respectively. Oral or intraperitoneal co‐administration of DGX did not notably change the liver/plasma ratio of ^14^C‐concentration (67–97 and 41–43 in male and female, respectively). In addition, liver/plasma ratios of S‐3100‐CA in DGX co‐administered mice were 89–139 and 50–56 in males and females, respectively. These results indicated that the transfer of S‐3100‐CA from plasma to liver was decreased by the inhibition of both Oatp1a1 and Oatp1a4 with RIF, but was unchanged in the presence of DGX, an Oatp1a4‐specific inhibitor. Taking into consideration this and previously published data about the expression levels of OATPs in mice, it was concluded that the most predominant isoform Oatp1a1 was the main contributor to hepatic uptake of S‐3100‐CA in mice.

Though oral co‐administration of DGX did not decrease the liver/plasma ratio of S‐3100‐CA, it did significantly decrease concentrations of S‐3100‐CA in plasma, liver, and kidney, while intraperitoneal co‐administration of DGX did not change these parameters. This result indicates that oral administration of DGX might decrease oral absorption of epyrifenacil. DGX is also known as a P‐glycoprotein substrate and it is reported that the absorption of DGX is competitively inhibited by other P‐glycoprotein substrates.^30^ Thus, in this study, DGX might inhibit the intestinal absorption of epyrifenacil,however, the effect of DGX interaction with epyrifenacil on intestinal absorption via P‐glycoprotein was not investigated in this study.

From the observations above, it was suggested that species differences in hepatic uptake between humans and mice were also due to the different expression levels of OATPs. In the previous literature, the plasma membrane protein levels of hepatic OATPs were higher in mouse liver (Oatp1a1: 42.9 ± 2.57 fmol/μg protein, Oatp1a4: 1.65 ± 0.14 fmol/μg protein)^13^ than in human livers (OATP1B1: 2.74 ± 3.67 fmol/μg protein, OATP1B3: 1.70 ± 0.45 fmol/μg protein, OATP2B1: 0.463 ± 0.872 fmol/μg protein).^15^ In addition, the total membrane protein levels of OATPs were higher in rats (3.91–6.43 fmol/μg protein) than in humans (1.1–2.0 fmol/μg protein).^16^ Taken together, the data indicate that the species differences in the hepatic uptake of S‐3100‐CA between mice and humans is not caused by differences in the selectivity toward OATP isoforms but rather to differences in the expression levels of OATPs in the liver. Based on the knowledge obtained in the current research about the active transport, further quantitative evaluation such as PBPK modeling is anticipated to complete the human safety assessment of S‐3100‐CA.

In conclusion, this study revealed that hepatic uptake of S‐3100‐CA was mediated by OATPs, and gender differences in rodents and species differences between rodents and humans in the hepatocyte uptake of S‐3100‐CA were not due to differences in selectivity toward OATP isoforms but rather to differences in protein expression levels. OATP expressing cells were successfully constructed and used to confirm that S‐3100‐CA was taken up by all OATPs examined in this study, suggesting that uptake of S‐3100‐CA was similar among OATP isoforms. When epyrifenacil was administered to mice, S‐3100‐CA was highly distributed to the liver and there was a gender difference. In vivo experiments using OATP inhibitors revealed that the most predominant OATP isoform in mice, Oatp1a1, contributes to the hepatic transport of S‐3100‐CA, and the gender difference in the expression levels of the isoform was consistent with the gender difference in the hepatic uptake of S‐3100‐CA in mice. These results suggest that the OATP expression levels should be the dominant determinant of the hepatic uptake of S‐3100‐CA. The significantly lower hepatic uptake in humans than in mice observed in the previously performed in vitro hepatic uptake study could be explained in the same way, that is, by much lower total expression levels of OATPs in humans than in mice. Consequently, the low hepatic uptake of S‐3100‐CA in humans resulting in the lower concern with its hepatotoxicity in humans than in mice was mechanistically supported by this research.

## DISCLOSURE

This work received no external funding. The author declares that there is no conflict of interest.

## AUTHOR CONTRIBUTIONS

Participated in research design: KS, TK, TH, JA, SK. Conducted experiments: KS, TK. Performed data analysis: KS, TK. Wrote or contributed to the writing of the manuscript: KS, TK, TH, JA, SK.

## ETHICS STATEMENT

All animal experiments were conducted in accordance with the Guidance for the Care and Use of Laboratory Animals in our laboratory, which corresponds to the Guidelines for Proper Conduct of Animal Experiments (Science Council of Japan). This manuscript is not published or submitted elsewhere.

## Data Availability

The data that support the findings of this study are available from the corresponding author upon reasonable request.

## References

[1] Dayan FE , Duke SO . Protoporphyrinogen oxidase‐inhibiting herbicides. In Krieger R , ed. Hayes’ Handbook of Pesticide Toxicology. Academic Press; 2010:1733‐1751. doi:10.1016/B978-0-12-374367-1.00081-1. https://www.sciencedirect.com/science/article/pii/B9780123743671000811?via%3Dihub

[2] Wakabayashi K , Böger P . Target sites for herbicides: entering the 21st century. Pest Manag Sci. 2002;58:1149‐1154. doi:10.1002/ps.560. https://onlinelibrary.wiley.com/doi/10.1002/ps.560 12449535

[3] Jacobs JM , Sinclair PR , Gorman N , et al. Effects of diphenyl ether herbicides on porphyrin accumulation by cultured hepatocytes. J Biochem Toxicol. 1992;7:87‐95. doi:10.1002/jbt.2570070206. https://onlinelibrary.wiley.com/doi/10.1002/jbt.2570070206 1404247

[4] Krijt J , Psenák O , Vokurka M , Chlumská A , Fakan F . Experimental hepatic uroporphyria induced by the diphenyl‐ether herbicide fomesafen in male DBA/2 mice. Toxicol Appl Pharmacol. 2003;189:28‐38. doi:10.1016/S0041-008X(03)00087-5. https://www.sciencedirect.com/science/article/pii/S0041008X03000875?via%3Dihub 12758057

[5] Leet JK , Hipszer RA , Volz DC . Butafenacil: a positive control for identifying anemia‐ and variegate porphyria‐inducing chemicals. Toxicol Rep. 2015;2:976‐983. doi:10.1016/j.toxrep.2015.07.006. https://www.sciencedirect.com/science/article/pii/S2214750015300263?via%3Dihub 28962437 PMC5598413

[6] Matsunaga K , Fukunaga S , Abe J , Takeuchi H , Kitamoto S , Tomigahara Y . Comparative hepatotoxicity of a herbicide, epyrifenacil, in humans and rodents by comparing the dynamics and kinetics of its causal metabolite. J Pestic Sci. 2021. in press. 10.1584/jpestics.D21-026 PMC8640676 34908893

[7] Kato Y , Kuge K , Kusuhara H , Meier PJ , Sugiyama Y . Gender difference in the urinary excretion of organic anions in rats. J Pharmacol Exp Ther. 2002;302:483‐489. doi:10.1124/jpet.102.033878. https://jpet.aspetjournals.org/content/302/2/483 12130705

[8] Kim SJ , Heo SH , Lee DS , Hwang IG , Lee YB , Cho HY . Gender differences in pharmacokinetics and tissue distribution of 3 perfluoroalkyl and polyfluoroalkyl substances in rats. Food Chem Toxicol. 2016;97:243‐255. doi:10.1016/j.fct.2016.09.017. https://www.sciencedirect.com/science/article/abs/pii/S0278691516303325?via%3Dihub 27637925

[9] Riccardi K , Lin J , Li Z , et al. Novel method to predict in vivo liver‐to‐plasma K_puu_ for OATP substrates using suspension hepatocytes. Drug Metab Dispos. 2017;45:576‐580. doi:10.1124/dmd.116.074575. https://dmd.aspetjournals.org/content/45/5/576 28258068

[10] Evers R , Piquette‐Miller M , Polli JW , et al.; International Transporter Consortium . Disease‐associated changes in drug transporters may impact the pharmacokinetics and/or toxicity of drugs: a white paper from the international transporter consortium. Clin Pharmacol Ther. 2018;104:900‐915. doi:10.1002/cpt.1115. https://ascpt.onlinelibrary.wiley.com/doi/10.1002/cpt.1115 29756222 PMC6424581

[11] Kalliokoski A , Niemi M . Impact of OATP transporters on pharmacokinetics. Brit J Pharmacol. 2009;158:693‐705. doi:10.1111/j.1476-5381.2009.00430.x. https://bpspubs.onlinelibrary.wiley.com/doi/10.1111/j.1476‐5381.2009.00430.x 19785645 PMC2765590

[12] Shugarts S , Benet LZ . The role of transporters in the pharmacokinetics of orally administered drugs. Pharm Res. 2009;26:2039‐2054. doi:10.1007/s11095-009-9924-0. https://link.springer.com/article/10.1007%2Fs11095‐009‐9924‐0 19568696 PMC2719753

[13] Kamiie J , Ohtsuki S , Iwase R , et al. Quantitative atlas of membrane transporter proteins: development and application of a highly sensitive simultaneous LC/MS/MS method combined with novel in‐silico peptide selection criteria. Pharm Res. 2008;25:1469‐1483. doi:10.1007/s11095-008-9532-4. https://link.springer.com/article/10.1007%2Fs11095‐008‐9532‐4 18219561

[14] Cheng X , Maher J , Chen C , Klaassen CD . Tissue distribution and ontogeny of mouse organic anion transporting polypeptides (Oatps). Drug Metab Dispos. 2005;33:1062‐1073. doi:10.1124/dmd.105.003640. https://dmd.aspetjournals.org/content/33/7/1062.short 15843488

[15] Ohtsuki S , Schaefer O , Kawakami H , et al. Simultaneous absolute protein quantification of transporters, cytochromes P450, and UDP‐glucuronosyltransferases as a novel approach for the characterization of individual human liver: comparison with mRNA levels and activities. Drug Metab Dispos. 2012;40:83‐92. doi:10.1124/dmd.111.042259. https://dmd.aspetjournals.org/content/40/1/83 21994437

[16] Wang L , Prasad B , Salphati L , et al. Interspecies variability in expression of hepatobiliary transporters across human, dog, monkey, and rat as determined by quantitative proteomics. Drug Metab Dispos. 2015;43:367‐374. doi:10.1124/dmd.114.061580. https://dmd.aspetjournals.org/content/43/3/367 25534768

[17] Eckhardt U , Schroeder A , Stieger B , et al. Polyspecific substrate uptake by the hepatic organic anion transporter Oatp1 in stably transfected CHO cells. Am J Physiol. 1999;276:G1037‐G1042. doi:10.1152/ajpgi.1999.276.4.G1037. https://journals.physiology.org/doi/full/10.1152/ajpgi.1999.276.4.G1037 10198348

[18] Gong L , Aranibar N , Han YH , et al. Characterization of organic anion‐transporting polypeptide (Oatp) 1a1 and 1a4 null mice reveals altered transport function and urinary metabolomic profiles. Toxicol Sci. 2011;122:587‐597. doi:10.1093/toxsci/kfr114. https://academic.oup.com/toxsci/article/122/2/587/1677312 21561886

[19] Hagenbuch B , Adler ID , Schmid TE . Molecular cloning and functional characterization of the mouse organic‐anion‐transporting polypeptide 1 (Oatp1) and mapping of the gene to chromosome X. Biochem J. 2000;345:115‐120. doi:10.1042/bj3450115. https://portlandpress.com/biochemj/article‐abstract/345/1/115/34817/Molecular‐cloning‐and‐functional‐characterization?redirectedFrom=fulltext 10600646 PMC1220737

[20] Izumi S , Nozaki Y , Komori T , et al. Substrate‐dependent inhibition of organic anion transporting polypeptide 1B1: comparative analysis with prototypical probe substrates estradiol‐17*β*‐glucuronide, estrone‐3‐sulfate, and sulfobromophthalein. Drug Metab Dispos. 2013;41:1859‐1866. doi:10.1124/dmd.113.052290. https://dmd.aspetjournals.org/content/41/10/1859 23920221

[21] Medwid S , Li MMJ , Knauer MJ , et al. Fexofenadine and rosuvastatin pharmacokinetics in mice with targeted disruption of organic anion transporting polypeptide 2B1. Drug Metab Dispos. 2019;47:832‐842. doi:10.1124/dmd.119.087619. https://dmd.aspetjournals.org/content/47/8/832 31123035

[22] Meyer zu Schwabedissen HE , Ware JA , Tirona RG , Kim RB . Identification, expression, and functional characterization of full‐length and splice variants of murine organic anion transporting polypeptide 1b2. Mol Pharm. 2009;6:1790‐1797. doi:10.1021/mp900030w. https://pubs.acs.org/doi/10.1021/mp900030w 19400585

[23] Ugele B , Bahn A , Rex‐Haffner M . Functional differences in steroid sulfate uptake of organic anion transporter 4 (OAT4) and organic anion transporting polypeptide 2B1 (OATP2B1) in human placenta. J Steroid Biochem Mol Biol. 2008;111:1‐6. doi:10.1016/j.jsbmb.2008.04.001. https://www.sciencedirect.com/science/article/abs/pii/S0960076008000873?via%3Dihub 18501590

[24] van Montfoort JE , Schmid TE , Adler ID , Meier PJ , Hagenbuch B . Functional characterization of the mouse organic‐anion‐transporting polypeptide 2. Biochim Biophys Acta. 2002;1564:183‐188. doi:10.1016/S0005-2736(02)00445-5. https://www.sciencedirect.com/science/article/pii/S0005273602004455?via%3Dihub 12101011

[25] Yang CH , Glover KP , Han X . Organic anion transporting polypeptide (Oatp) 1a1‐mediated perfluorooctanoate transport and evidence for a renal reabsorption mechanism of Oatp1a1 in renal elimination of perfluorocarboxylates in rats. Toxicol Lett. 2009;190:163‐171. doi:10.1016/j.toxlet.2009.07.011. https://www.sciencedirect.com/science/article/pii/S0378427409012247?via%3Dihub 19616083

[26] Grube M , Reuther S , Meyer zu Schwabedissen H , et al. Organic anion transporting polypeptide 2B1 and breast cancer resistance protein interact in the transepithelial transport of steroid sulfates in human placenta. Drug Metab Dispos. 2007;35:30‐35. doi:10.1124/dmd.106.011411. https://dmd.aspetjournals.org/content/35/1/30 17020956

[27] Miyajima M , Kusuhara H , Fujishima M , Adachi Y , Sugiyama Y . Organic anion transporter 3 mediates the efflux transport of an amphipathic organic anion, dehydroepiandrosterone sulfate, across the blood‐brain barrier in mice. Drug Metab Dispos. 2011;39:814‐819. doi:10.1124/dmd.110.036863. https://dmd.aspetjournals.org/content/39/5/814 21325432

[28] Tamai I , Nozawa T , Koshida M , Nezu J , Sai Y , Tsuji A . Functional characterization of human organic anion transporting polypeptide B (OATP‐B) in comparison with liver‐specific OATP‐C. Pharm Res. 2001;18:1262‐1269. doi:10.1023/A:1013077609227. https://link.springer.com/article/10.1023%2FA%3A1013077609227 11683238

[29] Ishida K , Ullah M , Tóth B , Juhasz V , Unadkat JD . Transport kinetics, selective inhibition, and successful prediction of in vivo inhibition of rat hepatic organic anion transporting polypeptides. Drug Metab Dispos. 2018;46:1251‐1258. doi:10.1124/dmd.118.080770. https://dmd.aspetjournals.org/content/46/9/1251 29891589

[30] Lumen AA , Li L , Li J , et al. Transport inhibition of digoxin using several common P‐gp expressing cell lines is not necessarily reporting only on inhibitor binding to P‐gp. PLoS One. 2013;8:e69394. doi:10.1371/journal.pone.0069394. https://journals.plos.org/plosone/article?id=10.1371/journal.pone.0069394 23976943 PMC3745465

